# [Retracted] miR‑141‑3p suppresses proliferation and promotes apoptosis by targeting GLI2 in osteosarcoma cells

**DOI:** 10.3892/or.2023.8576

**Published:** 2023-05-30

**Authors:** Nan Wang, Pengcheng Li, Wei Liu, Ningning Wang, Zhi Lu, Jianzhou Feng, Xiandong Zeng, Jiujie Yang, Yong Wang, Wei Zhao

Oncol Rep 39: 747–754, 2018; DOI: 10.3892/or.2017.6150

Following the publication of the above article, a concerned reader drew to the Editor's attention that, in the above paper, they had identified multiple instances of overlapping data panels comparing the TUNEL assay data shown in Fig. 2C and D on p. 750 with that shown in Fig. 4C on p. 752, suggesting that data purportedly showing results obtained under different experimental conditions had been derived from a smaller number of original sources. Upon informing the authors about this matter, they consulted their original data and requested a corrigendum to take account of the overlapping data in Figs. 2 and 4; however, given the number of instances of overlapping data panels that were identified comparing these two figures, the Editor of *Oncology Reports* has decided that this article should be retracted from the publication owing to a lack of overall confidence in the presented data. Upon informing the authors of this decision, they did not accepted the decision to retract this article. The Editor apologizes to the readership for any inconvenience resulting from the retraction of this article.

